# Racialized Black–White Economic Segregation and Major Chain Yoga Studio Locations in Major U.S. Metropolitan Areas

**DOI:** 10.1016/j.focus.2025.100417

**Published:** 2025-08-22

**Authors:** Kevin Y. Xu, Ruth Ling, Devin E. Banks, Benson S. Ku

**Affiliations:** 1 Division of Addiction Science, Prevention, and Treatment Research, Department of Psychiatry, Washington University School of Medicine, St. Louis, Missouri; 2 Center for the Study of Race, Ethnicity & Equity, Washington University in St. Louis, St. Louis, Missouri; 3 Washington University School of Public Health, St. Louis, Missouri; 4 Department of Psychiatry and Behavioral Sciences, Emory University School of Medicine, Atlanta, Georgia

**Keywords:** Yoga, health disparities, racism, socioeconomic factors, income, racial segregation

## Abstract

**Introduction:**

Despite yoga’s benefits and popularity, there is concern for racial inequities in yoga access and participation that have not been empirically studied at the population level. Against this background, the objective of this study was to examine the geographic distribution of major chain yoga franchises in relation to racialized Black–White economic segregation across the U.S.

**Methods:**

The authors extracted census tracts and 5-digit ZIP codes (ZIP5) for >500 major chain yoga franchises in the U.S. and estimated the Index of Concentration at the Extremes (ICE_Race[Black–White]-Income_), a measure of racialized Black–White economic segregation. ICE_Race(Black–White)-Income_ is a continuous variable ranging from −1 to +1, categorized into quintiles (−1=maximum deprivation; +1=maximum privilege).

**Results:**

Study findings revealed that <10% of studios were situated in census tracts and ZIP5s with a proportion of Black residents exceeding 13%. Less than 25% of studios were situated in census tracts and ZIP5s with household incomes below the 2022 national median ($74,580). Across all census tracts and ZIP5s, the mean value for ICE_Race(Black–White)-Income_ fell in the highest quintile for racialized Black–White economic segregation (ICE_Race[Black–White]-Income_=0.37).

**Conclusions:**

Major yoga franchises are situated in census tracts and ZIP codes that fall within the highest quintile of racialized Black–White economic segregation in the U.S.

## Highlights

• Census tracts and ZIP codes of 546 chain yoga studios in the U.S. were extracted.
• The authors analyzed the racialized Black–White economic segregation surrounding studios.
• Most studios are in areas within the highest quintile of Black–White economic segregation.
• Clinicians recommending yoga to patients should be mindful of access barriers.

## INTRODUCTION

Yoga is billed as an important modality for physical and mental health promotion, and its potential benefits include decreased anxiety, burnout, and reduced distress in the setting of chronic illnesses.^1^, ^2^, ^3^, ^4^, ^5^ Over 25% of physicians in the U.S. have reported recommending yoga to their patients.^6^ Nearly 50% of yoga practitioners in the U.S. report that a medical professional recommended yoga for health promotion.^7^ The American Psychiatric Association (APA), which has made Lifestyle Psychiatry the theme of its 2025 Annual Meeting, recently published guidance, in the APA-affiliated *Psychiatric News*, for mental health clinicians to integrate yoga practice, such as community yoga classes, into psychiatric treatment plans.^8^ The mental health benefits of yoga are also featured in the APA’s patient and family education materials, noting that yoga may “change the structure and function in the brain regions, including significant changes in areas involved in emotion regulation and stress.”^9^

Although yoga is typically associated with body poses and breath work, it also encompasses value-based practices (i.e., meditative absorption, self-discipline). Because yoga’s physicality and intellectual frameworks can be challenging for new practitioners to grasp, structured programming at yoga studios may help increase yoga’s accessibility.^10^ Structured programming may also foster social connection, community building, and collective healing that are central to yoga’s philosophy.^8^ National surveys suggest that 50% of people in the U.S. practice yoga outside their homes through studios, fitness centers, and athletic clubs.^11^

In recent years, yoga studios have been rapidly adopting corporate chain formats that now have franchises in the majority of the U.S.^12^, ^13^, ^14^ Although yoga, as it known today, has its roots in Asian and African healing traditions, there is concern that the rapidly expanding franchise studios in the U.S. are not accessible to racially, culturally, and socioeconomically diverse communities. The uptake of yoga practice has been reported to be higher in White populations than in racially minoritized populations in the U.S.,^15^^,^^16^ and among non-White communities, yoga studios have sometimes gained a reputation for being limited to the privileged few.^17^, ^18^, ^19^

Given that there is an increasingly extensive body of research illustrating the exclusivity of yoga studios in metropolitan areas with high Black–White residential segregation,^20^, ^21^, ^22^, ^23^ this preliminary cross-sectional ecologic study examines how racialized Black–White economic segregation may inform access to major yoga franchises in major U.S. metropolitan areas.

## METHODS

### Study Sample

The authors conducted a cross-sectional ecologic analysis using publicly available data. The authors extracted the census tracts and 5-digit ZIP codes (ZIP5) in the U.S. for all locations of 7 large chain yoga studios. The extracted studios spanned 38 U.S. states and captured the 30 largest American metropolitan statistical areas. Census tracts and ZIP5s were linked to the American Community Survey (ACS) (5-year estimates) to describe the racial and socioeconomic characteristics of each studio’s location. Detailed definitions of ZIP5s and the ACS are in the Appendix Table 1 (available online).

### Measures

The primary outcome variable was the Index of Concentration at the Extremes (ICE) (ICE_Race[Black–White]-Income_), a measure of racialized Black–White economic segregation,^24^^,^^25^ for each studio’s location. The authors estimated ICE_Race–Income_ by both census tract and ZIP5s. ICE_Race(Black–White)-Income_ measures the concentration of privileged versus deprived groups in a census tract or ZIP5 and is a promising measure for studying how structural racism and residential segregation may influence health outcomes. ICE_Race(Black–White)-Income_ is computed by subtracting the number of White persons at the highest quintile of income from the number of Black persons at the lowest quintile of income, divided by the total population in a census tract or ZIP5. ICE_Race(Black–White)-Income_ is a continuous variable ranging from −1 to 1, categorized into quintiles, with −1 reflecting maximum deprivation and 1 reflecting maximum privilege.

### Statistical Analysis

The authors compiled the addresses of 546 studios representing 7 large chain franchises (YogaSix, CorePower, Baptiste, Yoga Box, Yoga Pod, Hot 8, and Real Hot), allowing the extraction of ZIP5s and census tracts that were linked to publicly available ACS data. One studio was situated in a populated ZIP code but unpopulated census tract, culminating in 515 included tracts and 516 ZIP codes. At the time of data extraction (November 2023), extracted studios spanned all U.S. states except Alaska, Alabama, Delaware, Maine, Montana, Nebraska, New Hampshire, North Dakota, Rhode Island, South Dakota, West Virginia, and Wyoming. The authors confirmed that all 30 of the largest metropolitan statistical areas in the U.S. were captured across franchises. After extracting data from the ACS (ACSDT5Y2022.B02001 for census tracts and ACSDP5Y2021.DP05 for ZIP codes) on the median household income and racial and ethnic composition,^26^ the authors computed the ICE_Race(Black–White)-Income_ using the following formula: ICE_Race(Black–White)-Income_= (A*_i_*-P*_i_*)/T*_i_*, where A*_i_* represents the number of privileged persons in census tract (highest quintile of income, White [of any ethnicity]), P_i_ represents the number of socioeconomically deprived people in census tract (lowest quintile of income, Black [of any ethnicity]), and T*_i_* represents the total population in census tract *i* with known income and race. Although this formula is conducted at the level of the census tract, ICE_Race(Black–White)-Income_ can also be computed at the level of 5-digit ZIP codes. Statistical code and formulas for the calculation of ICE_Race(Black–White)-Income_ (using ACS data) are publicly available.^27^ For the present analysis, ZIP5s were linked to ACSDP5Y2021.DP05, and census tracts were linked to ACSDT5Y2022.B02001.

ICE_Race(Black–White)-Income_ is scaled from –1 to +1. –1 indicates that 100% of the population in the given area is concentrated in the most deprived group (Black [of any ethnicity], lowest quintile of income). +1 means that 100% of the population is concentrated in the most privileged group (White [of any ethnicity], highest quintile of income). For reference, the quintiles for ICE_Race (Black–White)-Income_ across all of the U.S. ZIP5s (2014–2018, ACS 5-year estimates^24^^,^^25^) are estimated to be the following: first quintile= −0.39, −0.10; second quintile= −0.10, 0.02; third quintile=0.02, 0.14; fourth quintile=0.14, 0.29; and fifth quintile=0.29, 0.70.

Analyses were conducted using SAS 9.4 from November 1, 2023 to December 15, 2023. The study was reviewed by the Washington University Human Research Protection Office and was determined to be exempt research.

## RESULTS

**Figure 1:**
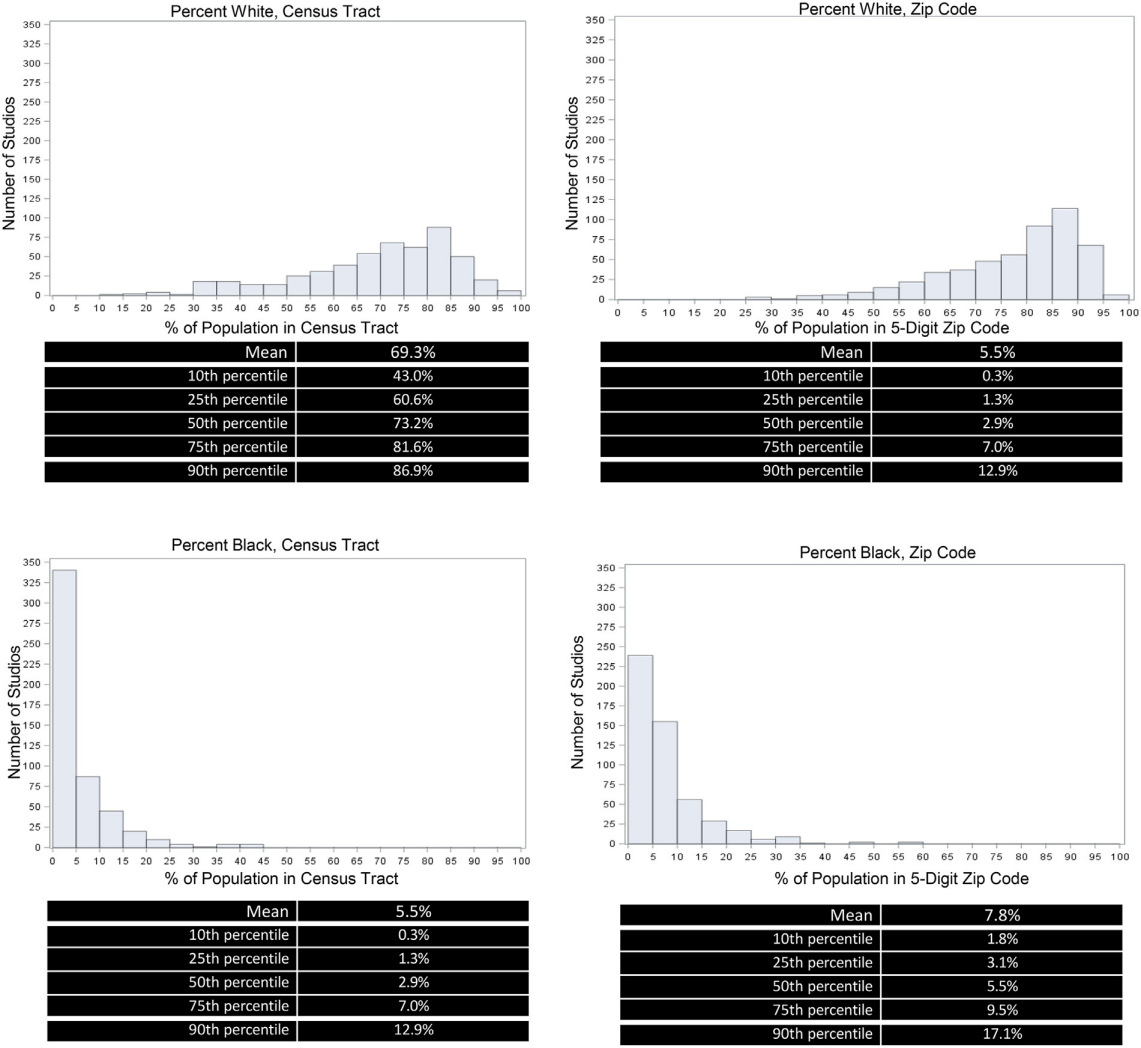
Racial and ethnic characteristics of census tracts and 5-digit ZIP codes containing major chain yoga studios in the U.S. *Note*: Histograms display the frequency distribution of studios across different percentages of White and Black populations in census tracts (left panels) and ZIP codes (right panels), with summary statistics showing percentile distributions.

The authors identified 546 studios representing 515 census tracts and 516 ZIP codes. As illustrated in Figure 1 and Appendix Figure 1 (available online), most studios were situated in predominantly White census tracts (median=73% White, 3% Black, 10.8% Hispanic, and 7.6% Asian American or Pacific Islander [AAPI]), with similar estimates for ZIP codes (median=81% White, 5.5% Black, 11.3% Hispanic, and 10.2% AAPI). Fewer than 10% of the studios were situated in census tracts with a proportion of Black residents exceeding 13%.

**Figure 2:**
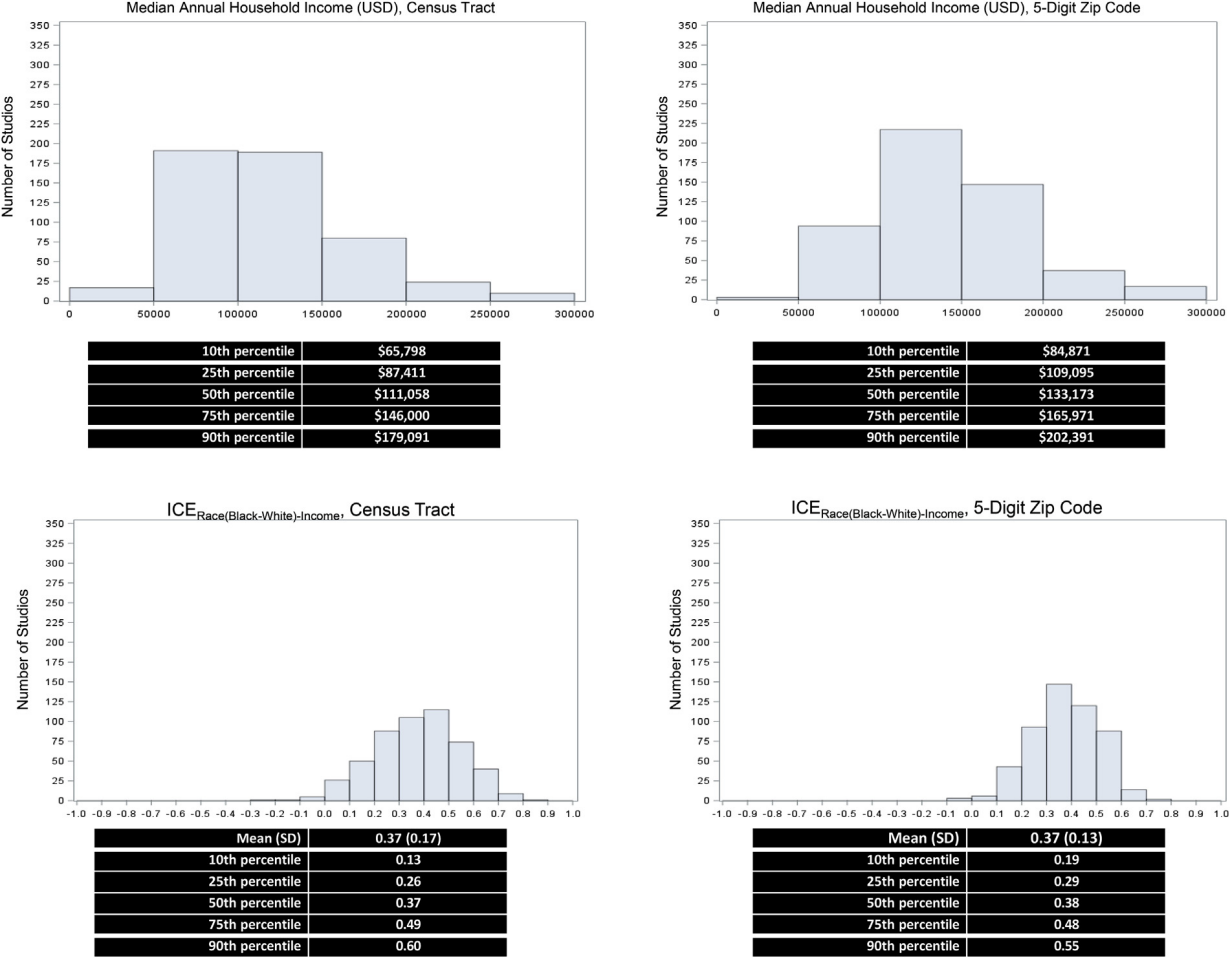
Income and racialized Black–White economic segregation containing major chain yoga studios in the U.S. *Note*: Histograms show the distribution of studios across median annual household income brackets (top panels) and ICE scores (bottom panels) for both census tracts and ZIP codes, with accompanying percentile summaries.ICE, Index of Concentration at the Extremes.

As shown in Figure 2, most studios were situated in the highest quintile of income census tracts (median annual household income=$111,058) and ZIP5s (median=$133,173); fewer than 25% of studios were situated in census tracts and ZIP5s with a household income below the 2022 national median ($74,580). The mean value for ICE_Race(Black–White)-Income_ was 0.37 (SD=0.17), with ICE_Race–Income_ scores ranging from 0.13 (10^th^ percentile in the sample of studios) to 0.60 (90^th^ percentile in the sample of studios). Similar estimates were obtained across ZIP codes (mean ICE_Race(Black–White)-Income_=0.37, SD=0.13).

## DISCUSSION

This investigation employed a cross-sectional ecologic design utilizing comprehensive linkage of major yoga franchise locations in major U.S. metropolitan areas to data from the ACS. Although these methods represent a thorough approach to understanding the geographic distribution of yoga access, they constitute an early stage of investigation that provides an empirical foundation for future research examining yoga accessibility and health equity. This analysis offers initial evidence that can inform subsequent studies incorporating longitudinal designs, individual-level data, and examination of causal mechanisms underlying the observed geographic patterns.

The analysis revealed notable patterns in studio distribution. Most chain yoga studios that the authors studied are situated in census tracts and ZIP codes that fall within the highest quintile of racialized Black–White economic segregation in the U.S. (top 20% of ICE_Race[Black–White]-Income_ across ZIP5s in the U.S.=0.29, 0.70).^25^ These results suggest that major yoga franchises are largely concentrated in racially and economically segregated White communities in the U.S., and they are seldom located in segregated Black communities that suffer from a legacy of concentrated poverty and disinvestment. Although it is increasingly common for clinicians to recommend yoga to their patients for health promotion,^7^^,^^9^ there is a dearth of research investigating disparities in access to yoga studios in the U.S. Given that convenience and cost are 2 of the most common traits for practitioners selecting studios,^11^^,^^28^ these data highlight geographic barriers to access that clinicians should account for when discussing yoga with their patients.

### Limitations

These findings provide some support for racialized Black–White economic segregation being part of the mechanism for inequities in yoga access and participation. Several methodologic considerations warrant acknowledgment. First, the cross-sectional and ecologic study design limits causal inference. Second, the ICE estimates are limited to Black–White segregation and cannot be generalized to other racial and ethnic groups (i.e., Hispanic, AAPI). Third, the location of the studio may not necessarily reflect the address of the resident. Finally, the data only represent large yoga franchises and do not generalize to the various ways yoga is practiced through community health centers, online classes, spiritual or religious customs, and independently owned businesses.^29^, ^30^, ^31^, ^32^ Despite these limitations, the findings are strengthened by the use of both ZIP5s and granular census tract–level data and have broad geographic coverage of the U.S. The findings suggest the need for targeted policy interventions and culturally adapted programming to address documented inequities in yoga access. Future research may consider examining community-based wellness initiatives and alternative yoga delivery models as potential mechanisms for improving access equity.^29^, ^30^, ^31^, ^32^ Professional organizations promoting lifestyle interventions may also benefit from incorporating structural and cultural considerations into their clinical guidance frameworks.^8^

## CONCLUSIONS

These findings have important implications for clinical practice and future research directions. Although this research does not comprehensively address every mode of access to yoga, it draws attention to how dominant figures of the yoga industry^14^ are physically positioned in places of Black–White racial divide and economic power. Studios are built to serve the people around them; the spatial concentration of major franchises raises important questions about the exclusivity of these studios and geographic and financial barriers to access.^13^ Furthermore, with smaller, local businesses struggling to compete with larger franchises,^13^^,^^33^ further research is needed to investigate the accessibility of yoga in marginalized communities.^29^ As clinicians are increasingly seen to attempt to integrate yoga into mental health treatment (i.e., recent efforts by the APA),^9^ this study data underscore the need for clinicians to be mindful of access barriers.

## Appendix Supplementary materials

Image, application 1

Image, application 2

## Footnotes

Supplementary material associated with this article can be found in the online version at doi:10.1016/j.focus.2025.100417.
