# Supplementary material for: Racialized Black–White Economic Segregation and Major Chain Yoga Studio Locations in Major U.S. Metropolitan Areas
Source: AJPM Focus. 2025 Aug 22;4(6):100417. doi: 10.1016/j.focus.2025.100417 (PMC12547918; doi:10.1016/j.focus.2025.100417)
Supplement: Supplementary file 1 [file mmc1.docx]

**Appendix Figure 1**: Racial and Ethnic Characteristics of Census Tracts and 5-Digit Zip Codes

Percent Hispanic, Census Tract


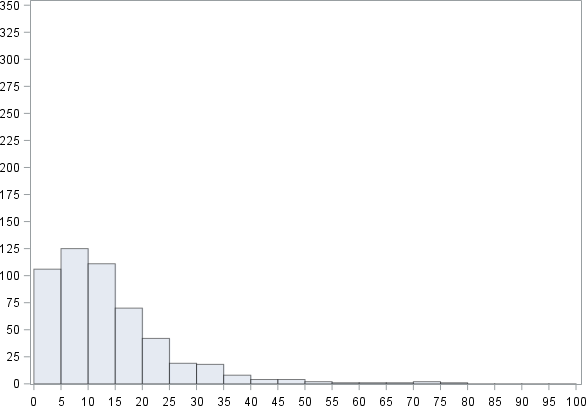


Number of Studios

% of Population in Census Tract

| Mean | 13.5% |
| --- | --- |
| 10th percentile | 3.0% |
| 25th percentile | 5.8% |
| 50th percentile | 10.8% |
| 75th percentile | 17.7% |
| 90th percentile | 26.7% |

Percent Hispanic, Zip Code


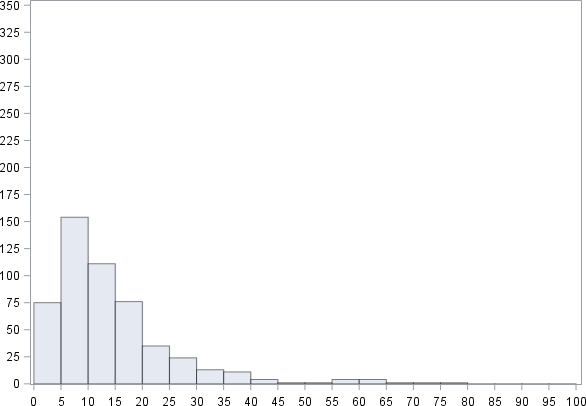


Number of Studios

% of Population in 5-Digit Zip Code

| Mean | 14.5% |
| --- | --- |
| 10th percentile | 4.0% |
| 25th percentile | 6.8% |
| 50th percentile | 11.3% |
| 75th percentile | 17.6% |
| 90th percentile | 27.5% |

Percent Asian American/Pacific Islander, Census Tract


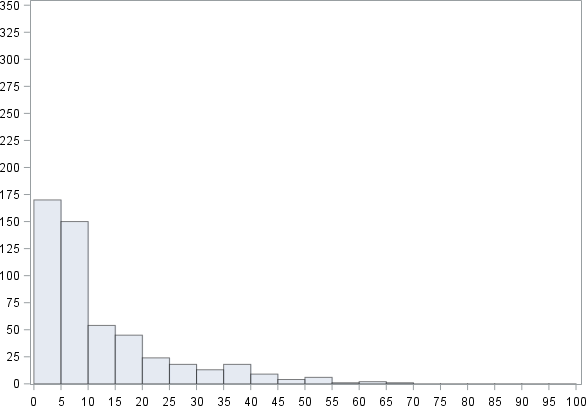


Number of Studios

% of Population in Census Tract

% of Population in Census Tract

| Mean | 14.5% |
| --- | --- |
| 10th percentile | 4.0% |
| 25th percentile | 6.8% |
| 50th percentile | 11.3% |
| 75th percentile | 17.6% |
| 90th percentile | 27.5% |

Percent Asian American/Pacific Islander, Zip Code


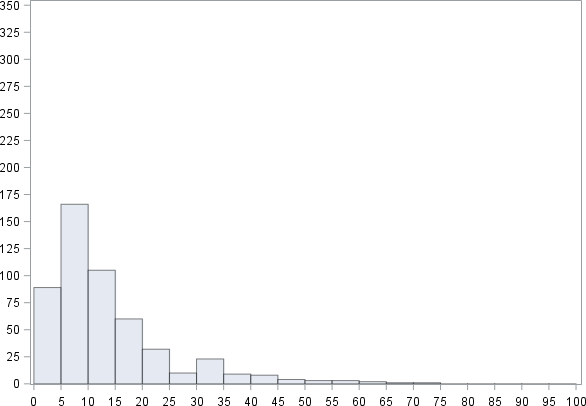


Number of Studios

% of Population in 5-Digit Zip Code

| Mean | 14.5% |
| --- | --- |
| 10th percentile | 4.0% |
| 25th percentile | 6.8% |
| 50th percentile | 11.3% |
| 75th percentile | 17.6% |
| 90th percentile | 27.5% |
