# Supplementary material for: Racialized Black–White Economic Segregation and Major Chain Yoga Studio Locations in Major U.S. Metropolitan Areas
Source: AJPM Focus. 2025 Aug 22;4(6):100417. doi: 10.1016/j.focus.2025.100417 (PMC12547918; doi:10.1016/j.focus.2025.100417)
Supplement: Supplementary file 2 [file mmc2.docx]

**Appendix Table 1**: Definitions of 5-Digit Zip Codes, the American Community Survey, and Index of Concentration at the Extremes

| ZIP5 | **ZIP Codes**: ZIP codes (Zone Improvement Plan codes) are postal codes used by the United States (U.S.) Postal Service to efficiently sort and deliver mail across the United States. ZIP codes divide the country into geographic regions for mail delivery purposes. These codes serve as important geographic identifiers in health research because they often correspond to meaningful community boundaries and can be linked to demographic and socioeconomic data from the U.S. Census Bureau.  ZIP5s refer specifically to five-digit ZIP codes, which represent the standard format for U.S. postal codes consisting of five numerical digits (e.g., 90210). While ZIP codes can be extended to include additional digits (ZIP+4 format with nine total digits), ZIP5s provide sufficient geographic granularity for population health research while maintaining adequate sample sizes for statistical analysis. Five-digit ZIP codes typically encompass populations ranging from a few hundred to several thousand residents, making them useful units for examining community-level health determinants and healthcare access patterns.  Reference: <https://faq.usps.com/s/article/ZIP-Code-The-Basics> |
| --- | --- |
| ACS | **ACS** (American Community Survey): The American Community Survey is a continuous demographic and socioeconomic data collection program conducted by the U.S. Census Bureau that replaced the long-form census questionnaire. The ACS collects detailed information about American communities every year rather than once every ten years, providing ongoing demographic, social, economic, and housing data for communities across the United States. This survey provides critical data on population characteristics including race, ethnicity, income, education, employment, and housing that researchers use to understand health disparities and community needs.  Reference: <https://www.census.gov/programs-surveys/acs/about.html> |
| ICE | **ICE** (Index of Concentration at the Extremes): The Index of Concentration at the Extremes is a measure of segregation and polarization that captures the concentration of privilege versus deprivation within a geographic area. Developed by Krieger and colleagues, ICE measures range from -1 to +1, where -1 indicates maximum concentration of the most deprived group and +1 indicates maximum concentration of the most privileged group. ICE can be calculated for various dimensions of inequality (racial, economic, or combined racialized economic measures) and has been increasingly used in public health research to understand how structural inequalities shape health outcomes and healthcare access patterns.  Reference: Krieger N, Waterman PD, Spasojevic J, Li W, Maduro G, Van Wye G. Public Health Monitoring of Privilege and Deprivation With the Index of Concentration at the Extremes. Am J Public Health. 2016 Feb;106(2):256-63. doi: 10.2105/AJPH.2015.302955. Epub 2015 Dec 21. PMID: 26691119; PMCID: PMC4815605. |
